# Methylmalonic acid, vitamin B12, renal function, and risk of all-cause mortality in the general population: results from the prospective Lifelines-MINUTHE study

**DOI:** 10.1186/s12916-020-01853-x

**Published:** 2020-12-10

**Authors:** Ineke J. Riphagen, Isidor Minović, Dion Groothof, Adrian Post, Manfred L. Eggersdorfer, Jenny E. Kootstra-Ros, Martin H. de Borst, Gerjan Navis, Frits A. J. Muskiet, Ido P. Kema, M. Rebecca Heiner-Fokkema, Stephan J. L. Bakker

**Affiliations:** 1 Department of Laboratory Medicine, University of Groningen, University Medical Center Groningen, P.O. Box 30.001, 9700 RB Groningen, The Netherlands; 2 Department of Internal Medicine, Division of Nephrology, University of Groningen, University Medical Center Groningen, Groningen, The Netherlands; 3 DSM Nutritional Products, Kaiseraugst, Switzerland

## Abstract

**Background:**

Methylmalonic acid (MMA) is best known for its use as a functional marker of vitamin B12 deficiency. However, MMA concentrations not only depend on adequate vitamin B12 status, but also relate to renal function and endogenous production of propionic acid. Hence, we aimed to investigate to what extent variation in MMA levels is explained by vitamin B12 and eGFR and whether MMA levels are associated with mortality if vitamin B12 and eGFR are taken into account.

**Methods:**

A total of 1533 individuals (aged 60–75 years, 50% male) were included from the Lifelines Cohort and Biobank Study. Individuals were included between 2006 and 2013, and the total follow-up time was 8.5 years.

**Results:**

Median [IQR] age of the study population was 65 [62–69] years, 50% was male. At baseline, median MMA concentration was 170 [138–216] nmol/L, vitamin B12 290 [224–362] pmol/L, and eGFR 84 [74–91] mL/min/1.73 m2. Log_2_ vitamin B12, log_2_ eGFR, age, and sex were significantly associated with log_2_ MMA in multivariable linear regression analyses (model *R*^2^ = 0.22). After a total follow-up time of 8.5 years, 72 individuals had died. Log_2_ MMA levels were significantly associated with mortality (hazard ratio [HR] 1.67 [95% CI 1.25–2.22], *P* < 0.001). Moreover, we found a significant interaction between MMA and eGFR with respect to mortality (*P*_interaction_ < 0.001).

**Conclusions:**

Only 22% of variation in MMA levels was explained by vitamin B12, eGFR, age, and sex, indicating that a large part of variation in MMA levels is attributable to other factors (e.g., catabolism, dietary components, or gut microbial production). Higher MMA levels are associated with an increased risk for mortality, independent of vitamin B12, eGFR, and sex. This association was more pronounced in individuals with impaired renal function.

## Background

Methylmalonic acid (MMA) is a small water-soluble organic acid that is currently best known for its use as a functional marker for vitamin B12 deficiency [1]. Vitamin B12 is an essential cofactor for L-methylmalonyl-CoA mutase, which converts methylmalonyl-CoA into succinyl-CoA [2]. In vitamin B12 deficiency, the activity of L-methylmalonyl-CoA mutase is impaired, which results in the conversion of methylmalonyl-CoA into MMA [2].

However, concentrations of MMA are not only elevated in vitamin B12 deficiency, but are also known to increase during renal dysfunction [3, 4]. Moreover, methylmalonyl-CoA is an intermediate in the metabolism of propionic acid (PA), which is produced as a result of beta-oxidation of odd-chain fatty acids and branched-chain amino acids, metabolism of cholesterol side-chains, and fermentation by colonic microbiota [5, 6].

Thus, circulating concentrations of MMA depend to a certain extent on plasma vitamin B12 level and renal function but may also reflect endogenous production of PA. Importantly, PA has been found to be highly toxic [7]. Studies with PA in isolated hepatocytes suggest that toxicity of PA is due to accumulation of propionyl-CoA and methylmalonyl-CoA [7]. Intracellular accumulation of propionyl-CoA and methylmalonyl-CoA was found to be associated with impairment of several hepatic metabolic pathways, including gluconeogenesis, ureagenesis, pyruvate oxidation, and fatty acid oxidation [7].

In the present study, we first investigated to what extent MMA levels are explained by plasma vitamin B12 and eGFR. The variance in circulating MMA concentrations that remains unexplained by plasma vitamin B12 and eGFR may, at least in part, reflect PA production. We also investigated whether circulating MMA concentrations are associated with an increased risk for mortality and whether this association is independent of or interdependent with plasma vitamin B12 or eGFR.

## Methods

### Study design and population

The LifeLines Cohort Study is a large ongoing observational population-based cohort study that investigates health and health-related behaviors of more than 167,000 individuals. A detailed description of the Lifelines Cohort Study can be found elsewhere [8, 9]. Participants were recruited from the three Northern provinces of the Netherlands between 2006 and 2013. In short, the first group of participants was recruited via local general practices. Participants could indicate whether family members were interested as well. In addition, individuals who were interested in the study had the possibility to register via an online self-registration. Individuals with insufficient knowledge of the Dutch language, with severe psychiatric or physical illness, and those with limited life expectancy (< 5 years) were excluded from the study. Participants completed several questionnaires, including topics such as the occurrence of diseases, general health, medication use, diet, physical activity, and personality. Participants were invited to the Lifelines Research sites for a comprehensive health assessment and to allow storage of biological samples, including plasma, serum, and 24-h urine samples in the biobank underlying the LifeLines cohort. All participants provided written consent. The Lifelines Cohort Study was conducted according to the principles of the Declaration of Helsinki and approved by the Medical ethical committee of the University Medical Center Groningen, the Netherlands (METc approval number 2007/152).

The present study included individuals of the LifeLines-MINUTHE (MIcroNUTrients and Health disparities in Elderly) subcohort of the LifeLines Cohort Study. This subcohort consists of 1605 individuals aged between 60 and 75 years, with available plasma, serum, and 24-h urine samples from the biobank of the LifeLines cohort. The 1605 individuals comprised 400 men and 403 women with low socioeconomic status (SES) and 402 men and 400 women with high SES. Since education is more differentiating than income in the Dutch population, the classification of SES was based on educational status. Low SES was defined as never been to school or elementary school only, or completed lower vocational or secondary schooling; high SES was defined as completed higher vocational schooling or education. In the present study, we included 1533 individuals with available MMA, vitamin B12, and eGFR measurements.

### Data collection and measurements

Data regarding demographics, education, smoking status, and general health were collected from self-administered questionnaires. Anthropometric measurements and blood pressure were measured by well-trained staff. BMI was calculated as weight (kg) divided by height squared (m^2^). Systolic and diastolic blood pressures were measured 10 times during a period of 10 min using an automated Dinamap Monitor (GE Healthcare, Freiburg, Germany). The average of the final three readings was used for each blood pressure parameter.

Blood samples were collected in a fasting state between 8.00 and 10.00 a.m. and subsequently transported to the Central Lifelines Laboratory in the University Medical Center Groningen. MMA was measured using LC-MS/MS. Vitamin B12 was measured using an electrochemiluminiscence immunoassay on a Roche Cobas chemistry analyzer (Roche, Mannheim, Germany). Serum creatinine (SCr) was measured via an enzymatic assay with colorimetric detection on a Roche Cobas chemistry analyzer (Roche, Mannheim, Germany). The creatinine-based CKD-EPI formula was used to obtain the estimated glomerular filtration rate (eGFR) [10]. Other laboratory measurements, including plasma total homocysteine, were assessed by commercially available assays on a Roche Cobas chemistry analyzer (Roche, Mannheim, Germany).

### Clinical endpoints

In the present study, we investigated the association of MMA with all-cause mortality. Data on mortality were obtained from the municipal register.

### Statistical analyses

Statistical analyses were performed using SPSS version 25 for Windows (IBM Corporation, Chicago, IL), STATA version 13.1 (StataCorp LP, TX, USA), and R version 3.5.2 (R Core Team (2017); R: A language and environment for statistical computing; R Foundation for Statistical Computing, Vienna, Austria; URL https://R-project.org/). Results were expressed as mean ± standard deviation (SD) or median (interquartile range) for normally and non-normally distributed data, respectively. Nominal data were presented as the total number of patients (percentage). A two-sided *P* < 0.05 was considered to indicate statistical significance.

Baseline characteristics are presented for the total study population and for tertiles of baseline MMA concentrations. *P* values for differences between tertiles were assessed using ANOVA for normally distributed data, Kruskal-Wallis test for skewed data, and the *χ*^2^ test for nominal data.

We used linear regression analyses to investigate the cross-sectional associations of MMA with vitamin B12, eGFR, and other parameters including SES. Logarithmic transformation of variables was used to fulfill criteria for linear regression analyses if necessary. First, univariable linear regression analyses were conducted. In addition, we tested for interactions between variables using multivariable linear regression analyses. Finally, multivariable linear regression models were developed using stepwise backward selection, without and with inclusion of the interaction term for vitamin B12 and eGFR (model 1 and model 2, resp.). Variable exclusion in the backward stepwise selection procedure was set at a *P* value of 0.2; the *P* value for subsequent variable inclusion was set to 0.05. Results for variables with a *P* value of > 0.2 in univariable and multivariable linear regression analyses were not shown. *R*^2^ and adjusted *R*^2^ values were obtained to assess the proportion of variability in the data accounted for by single variables and the multivariable models. The R package plot3D was used to depict the cross-sectional interaction between vitamin B12 and eGFR with MMA levels.

We used Cox regression analyses to investigate the prospective association of MMA with all-cause mortality. We applied a log_2_ transformation of MMA values so the hazard ratios were expressed as an increase in risk per doubling of baseline MMA values. Cox regression analyses were also used to test for interaction between MMA and eGFR. Various Cox regression models were built to adjust for possible confounders. The first model depicts the interaction between log_2_ MMA and eGFR for the risk of mortality; model 2 was adjusted for age and sex; and model 3 was additionally adjusted for SES, smoking, alcohol intake, BMI, SBP, vitamin B12, and use of vitamin. In secondary analyses, we investigated whether prospective associations for MMA were paralleled by prospective associations for plasma total homocysteine. Model 1A and model 1B depict the interaction between log_2_ total plasma homocysteine and eGFR with respect to mortality and the interaction between log_2_ MMA and eGFR with respect to mortality; model 2A and model 2B were fully adjusted for potential confounders, with additional adjustment for log_2_ MMA in case of the analysis for total plasma homocysteine (model 2A) and additional adjustment for log_2_ total plasma homocysteine in case of the analysis for MMA (model 2B); and model 3 was fully adjusted for potential confounders and included both the interaction between log_2_ total plasma homocysteine and eGFR with respect to mortality and the interaction between log_2_ MMA and eGFR with respect to mortality in one model. The assumption of proportional hazards was investigated by inspecting the Schoenfeld residuals. The R package plot3D was used to depict the interaction of MMA and eGFR in their association with mortality. As sensitivity analyses, we stratified Cox regression analyses for SES. In addition, we repeated Cox regression analyses after exclusion of subjects that used multivitamin or vitamin B supplements.

## Results

### Baseline characteristics

In this study, we included 1533 individuals. Baseline characteristics of the total study population and according to tertiles of MMA concentrations are presented in Table 1. At baseline, median MMA concentration was 170 (range 63–4638) nmol/L, median vitamin B12 concentration was 290 (range 64–1476) pmol/L), and median eGFR was 84 (range 19–109) mL/min/1.73 m^2^. A total of 104 individuals (7%) had an MMA level > 340 nmol/L, 52 individuals (3%) had a vitamin B12 level < 145 pmol/L, and 166 individuals (11%) had a vitamin B12 > 450 pmol/L. We found that age, low education, concentrations of vitamin B12, concentrations of total homocysteine, eGFR, and use of vitamin supplements were significantly different across tertiles of MMA (Table 1).

**Table 1:** Baseline characteristics of the study population (*n* = 1533)

	All subjects (***n*** = 1533)	Tertiles of MMA			***P*** value
		Tertile 1 (***n*** = 511)	Tertile 2 (***n*** = 511)	Tertile 3 (***n*** = 511)	
MMA (nmol/L)	170 (138–216)	≤ 148	148–196	≥ 196	–
Vitamin B12 (pmol/L)	290 (224–362)	332 (264–416)	295 (231–356)	240 (190–309)	< 0.001
eGFR (mL/min/1.73 m^2^)	84 (74–91)	86 (77–92)	83 (75–91)	81 (69–90)	< 0.001
**Demographics**					
Male sex (*n*, %)	769 (50)	254 (50)	252 (50)	263 (51)	0.8
Age (years)	65 (62–69)	64 (62–68)	65 (62–69)	65 (63–69)	< 0.001
Education					< 0.001
Low (*n*, %)	761 (50)	228 (45)	242 (47)	291 (57)	
High (*n*, %)	772 (50)	283 (55)	269 (53)	220 (43)	
Smoking (*n*, %)	183 (12)	55 (11)	62 (12)	66 (13)	0.6
Alcohol consumption					0.9
Non-drinker (*n*, %)	242 (16)	79 (15)	84 (16)	79 (15)	
≤ 1 drink/day (*n*, %)	552 (36)	185 (36)	190 (37)	177 (35)	
1–2 drinks/day (*n*, %)	309 (20)	114 (22)	101 (20)	94 (18)	
> 2 drinks/day (*n*, %)	174 (11)	63 (12)	61 (12)	50 (10)	
Diabetes (*n*, %)	156 (10)	58 (11)	42 (8)	56 (11)	0.2
History of CVD (*n*, %)	186 (12)	58 (11)	65 (13)	63 (12)	0.8
**Clinical measurements**					
BMI (kg/m^2^)	26.4 (24.1–29.4)	26.1 (24.1–29.1)	26.5 (24.0–29.3)	26.3 (24.1–29.8)	0.5
Systolic blood pressure (mmHg)	134 ± 18	134 ± 17	134 ± 17	135 ± 18	0.6
Diastolic blood pressure (mmHg)	75 ± 9	75 ± 9	75 ± 9	76 ± 10	0.2
**Laboratory parameters**					
Hb (mmol/L)	8.8 ± 0.7	8.8 ± 0.7	8.8 ± 0.7	8.8 ± 0.7	0.6
MCV (fL)	91 ± 4	91 ± 4	91 ± 4	91 ± 4	0.7
Total cholesterol-HDL ratio	3.5 (2.9–4.3)	3.5 (2.9–4.4)	3.5 (3.0–4.3)	3.5 (2.9–4.4)	0.9
Triglycerides (mmol/L)	1.1 (0.8–1.5)	1.1 (0.8–1.5)	1.1 (0.8–1.5)	1.1 (0.8–1.5)	0.4
Serum creatinine (μmol/L)	75 (66–85)	73 (64–84)	75 (66–84)	78 (67–88)	< 0.001
Total homocysteine (μmol/L)	13 (11–15)	12 (11–14)	13 (11–15)	14 (12–17)	< 0.001
**Medication**					
Vitamin supplements (*n*, %)	173 (11)	71 (14)	58 (11)	44 (9)	0.03

*BMI* body mass index, *eGFR* estimated glomerular filtration rate, *Hb* hemoglobin, *HDL* high-density lipoprotein, *MMA* methylmalonic acid

### Determinants of MMA

**Fig. 1:**
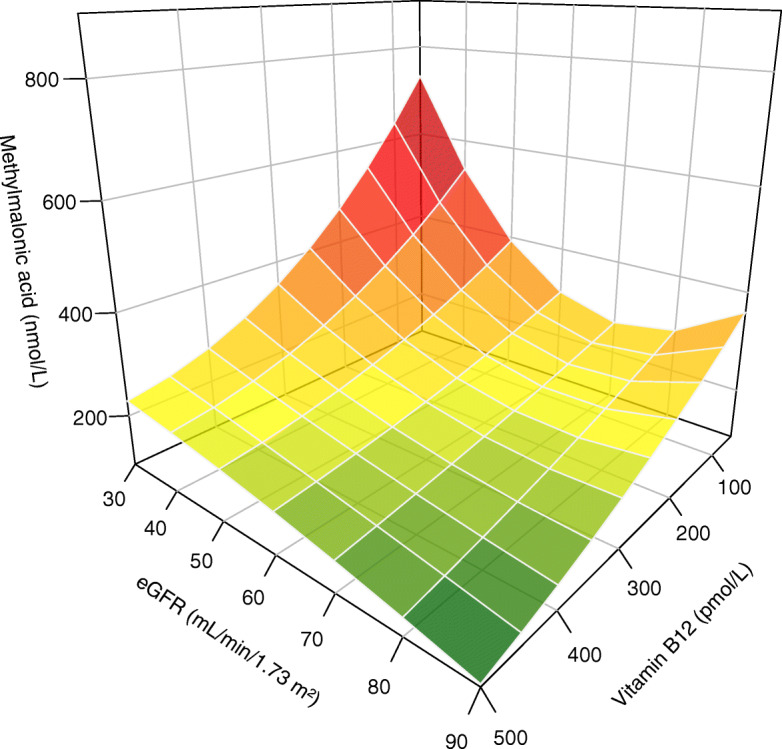
3D plot depicting the unadjusted cross-sectional association between methylmalonic acid, vitamin B12, and eGFR

Results of univariable and multivariable linear regression analyses are depicted in Table 2. We found a significant association of log_2_ vitamin B12, log_2_ eGFR, age, education, and use of vitamin supplements with log_2_ MMA in univariable linear regression analyses. *R*^2^ values were highest for log_2_ vitamin B12 (0.180) and log_2_ eGFR (0.036). Furthermore, we found a significant interaction between log_2_ vitamin B12 and log_2_ eGFR in multivariable linear regression analyses (*P*_interaction_ = 0.02). The *R*^2^ values for multivariable linear regression models 1 and 2 were 0.22 (Table 2). The cross-sectional association between vitamin B12, eGFR, and MMA is depicted in Fig. 1. As shown in Fig. 1, MMA concentrations are highest among subjects with the lowest vitamin B12 concentrations and lowest eGFR. These results did not materially change after the exclusion of subjects that used a multivitamin or vitamin B containing supplement (Additional files 1 and 2).

**Table 2:** Univariable and multivariable linear regression analyses for log_2_ MMA

	Univariable		Multivariable Model 1		Multivariable Model 2	
	Std ***b***	***P*** value	Std ***b***	***P*** value	Std ***b***	***P*** value
Log_2_ vitamin B12 (pmol/L)	− 0.424	< 0.001	− 0.434	< 0.001	1.131	0.08
Log_2_ eGFR (mL/min/1.73 m^2^)	− 0.189	< 0.001	− 0.162	< 0.001	0.689	0.048
Log_2_ vitamin B12 × log_2_ eGFR	–	–	–	–	− 1.796	0.01
**Demographics**						
Male sex	0.002	0.9	− 0.065	0.005	− 0.053	0.02
Age (years)	0.093	< 0.001	0.050	0.04	0.034	0.2
High education	− 0.099	< 0.001	–	–	− 0.030	0.2
**Medication**						
Use of vitamin supplements	− 0.069	0.007	–	–	–	–

Model 1: *R*^2^ = 0.22; adjusted *R*^2^ = 0.22

Model 2: *R*^2^ = 0.22; adjusted *R*^2^ = 0.21

*eGFR* estimated glomerular filtration rate, *MMA* methylmalonic acid

### MMA, eGFR, and mortality

**Fig. 2:**
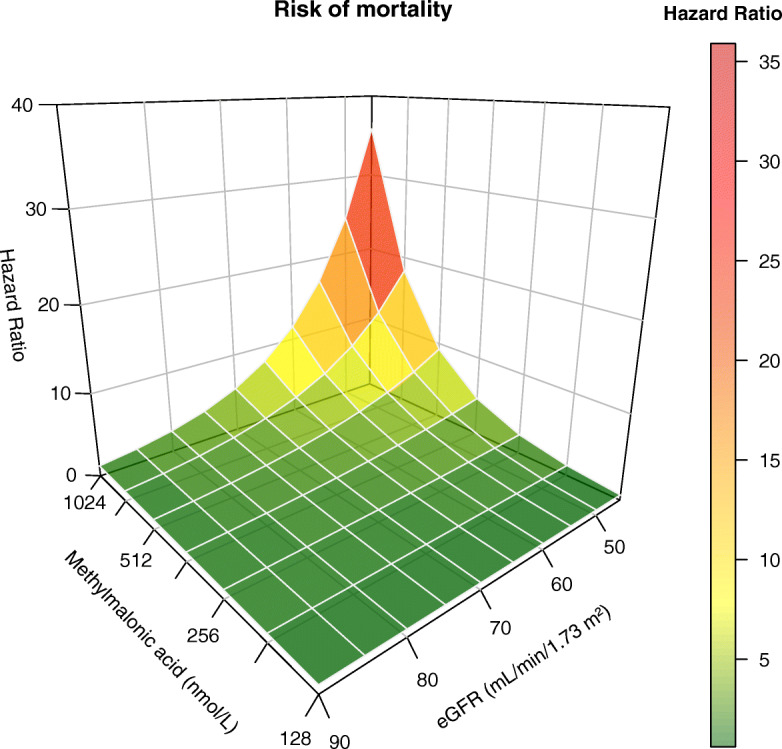
3D plot depicting the unadjusted interaction between methylmalonic acid and eGFR with all-cause mortality

After a total follow-up time of 8.5 years (median 5.3 [4.3–6.5] years), 72 subjects had died. In univariable Cox regression analyses, we found a significant association of log_2_ MMA with mortality (HR 1.67 (95% CI 1.25–2.22), *P* < 0.001) and eGFR (per 10 mL/min/1.73 m^2^) with mortality (0.73 (0.62–0.85), *P* < 0.001), while log_2_ vitamin B12 was not significantly associated with mortality (0.74 (0.49–1.11), *P* = 0.1). Moreover, we found a significant interaction between MMA and eGFR with respect to mortality, which remained significant independent of adjustment for potential confounders (Table 3). The interaction between MMA and eGFR with respect to mortality is depicted in Fig. 2. Figure 2 shows that higher MMA values as well as lower eGFR values are associated with an increased risk for all-cause mortality and that this mortality risk strongly increases when MMA levels increase and eGFR decreases.

**Table 3:** Prospective associations of log_2_ MMA, eGFR, and their interaction term, with all-cause mortality

	Model 1		Model 2		Model 3	
	HR (95% CI)	***P*** value	HR (95% CI)	***P*** value	HR (95% CI)	***P*** value
Log_2_ MMA (nmol/L)	13.33 (3.87–45.91)	< 0.001	15.24 (4.47–52.03)	< 0.001	11.48 (3.32–39.64)	< 0.001
eGFR (10 mL/min/m^2^)	7.36 (1.98–27.41)	0.003	9.79 (2.61–36.70)	0.001	7.57 (1.98–28.94)	0.003
Log_2_ MMA × eGFR	0.76 (0.65–0.89)	0.001	0.74 (0.64–0.87)	< 0.001	0.77 (0.65–0.90)	0.001

Model 1: log_2_ MMA, eGFR, log_2_ MMA × eGFR

Model 2: adjusted for age and sex

Model 3: as model 2 + SES, smoking, alcohol intake, BMI, SBP, vitamin B12, and use of vitamin supplements

*N*_events_/*n*_total_ = 72/1533

*BMI* body mass index, *eGFR* estimated glomerular filtration rate, *SBP* systolic blood pressure, *SES* socioeconomic status

In secondary analyses, we investigated whether these findings for MMA might be paralleled by similar associations for plasma total homocysteine. In these analyses, we found a similar interaction for log_2_ total plasma homocysteine and eGFR with respect to mortality as for log_2_ MMA and eGFR with respect to mortality, albeit somewhat weaker for the former interaction compared to the latter interaction (Additional file 3, model 1A and model 1B, resp.). In models in which the interactions were adjusted for potential confounders, we applied additional adjustment for log_2_ MMA in case of the analysis for total plasma homocysteine (Additional file 3, model 2A), and we applied additional adjustment for log_2_ total plasma homocysteine in case of the analysis for MMA (Additional file 3, model 2B). In these analyses, the results were also consistent with a parallel interaction for both parameters, albeit again somewhat weaker for the interaction between plasma total homocysteine and eGFR than for the interaction between MMA and eGFR. In further analyses, in which we included both interactions in one fully adjusted model, the interaction between log_2_ plasma total homocysteine and eGFR lost significance (0.98 (0.77–1.26), *P*_interaction_ = 0.9), while the interaction between log_2_ MMA and eGFR remained (0.79 (0.65–0.97), *P*_interaction_ = 0.03) (Additional file 3, model 3).

As sensitivity analyses, we stratified Cox regression analyses for SES. The results of these sensitivity analyses were not materially different from the Cox regression analyses in which we adjusted for SES (Additional file 4). Furthermore, analyses were repeated after exclusion of subjects that used multivitamin or vitamin B supplements at baseline. The results did not materially change after exclusion of subjects that used multivitamin or vitamin B supplements at baseline (Additional files 5 and 6).

## Discussion

In this study, we found that vitamin B12 and eGFR were significantly associated with MMA levels. However, only 22% of the variation in MMA values was explained by vitamin B12, eGFR, age, and sex, indicating that a large part of variation in MMA levels was attributable to factors other than vitamin B12 and eGFR. Furthermore, to the best of our knowledge, we are the first to demonstrate that elevated MMA levels are associated with an increased risk for mortality. We found a significant interaction between MMA and eGFR in relation to mortality, indicating that this association was more pronounced in individuals with impaired renal function. These findings are of particular interest since MMA is a potentially modifiable marker, not only through pharmacological intervention, but also through dietary interventions, and could therefore be an important tool for guiding the improvement of health and longevity in the general population.

In this study, we investigated the cross-sectional association of vitamin B12 and eGFR with MMA. In line with previous studies [2–4], we found that MMA levels are associated with vitamin B12 and eGFR and that MMA levels are highest in subjects with a combination of low vitamin B12 levels and impaired renal function. However, we found that only 22% of variation in circulating MMA concentration was explained by vitamin B12, eGFR, age, sex, and SES. This finding is in line with a previous study from Vogiatzoglou et al. that demonstrated that 16% of variation in MMA was explained by vitamin B12, plasma creatinine, and sex [2]. Furthermore, Vogiatzoglou et al. could also not identify factors other than age, plasma creatinine, vitamin B12, and sex that substantially influenced plasma MMA [2]. The addition of anthropometric measures, lifestyle, and dietary factors (i.e., dietary intake of food items such as meat and fish) did not substantially add to the explained variation of MMA (*R*^2^ = 0.167) [2].

As stated previously, MMA is an intermediate in the metabolism of PA [5, 6]. The main source of PA is catabolism of the amino acids valine, isoleucine, methionine, and threonine, and other pathways including catabolism of odd-chain fatty acids and cholesterol side-chains [11]. Furthermore, PA is also produced by anaerobic fermentation of carbohydrates and other compounds within the gut, from which it is absorbed into the portal circulation [11, 12]. Although there are wide individual variations in PA production from different sources, stable isotope studies in patients with propionic and methylmalonic acidemia indicate that approximately 50% of PA production is derived from amino acid catabolism, 25% from anaerobic fermentation in the gut, and 25% from catabolism of odd-chain fatty acids [11, 13, 14]. Thus, variation in plasma MMA concentrations in the absence of vitamin B12 deficiency or renal dysfunction may also result from increased production of PA and its metabolites or decreased renal clearance thereof.

Importantly, to the best of our knowledge, this study is the first to demonstrate that elevated MMA levels are associated with an increased risk for all-cause mortality. Moreover, we found that this association was more pronounced in individuals with impaired renal function. Since the present study is an observational, and not a mechanistic, study, we can only speculate on possible underlying pathophysiological mechanisms for this association. First, elevated MMA may be a marker of abnormal gut microbiota, in particular high PA bacteria. It is known that chronic kidney disease (CKD) itself, together with CKD-related changes in diet and medication, can induce changes in both the composition and metabolic activity of gut microbiota [15]. These CKD-related changes in microbiota may also affect metabolic and cardiovascular health by secretion of metabolites that favor insulin resistance, obesity, endothelial dysfunction, and cardiovascular aging [15], which may explain the increased mortality risk in individuals with impaired renal function. In addition, since a large part of PA production is derived from catabolism of amino acids and odd-chain fatty acids [11, 13, 14], catabolism related to chronic diseases may also be a possible underlying mechanism [16]. It is known that catabolism is related with an increased morbidity and mortality risk in CKD [17, 18], which also might be a possible explanation for the strongly increased mortality risk in individuals with impaired renal function. In secondary analyses, we found that the interaction between MMA and eGFR with respect to mortality was paralleled by a similar, albeit less strong, interaction between plasma total homocysteine and eGFR with respect to mortality, which is suggestive of parallelism in mechanisms underlying prospective associations of MMA and plasma total homocysteine with respect to mortality.

Some limitations of the present study need to be addressed. First, given the unavailability of PA measurements, we were not able to investigate the association between MMA and PA levels. Additional studies are needed to investigate the association between MMA and PA and other potential factors influencing MMA levels. Second, given the observational nature of this study, it is impossible to draw a definite conclusion about the causality of the association of MMA with mortality. In addition, given the observational nature, we can only speculate on potential pathophysiological pathways. Further studies are needed to investigate whether the association between MMA and mortality is causal and to provide more insight in the underlying pathophysiological pathways, especially in subjects with chronic kidney disease. Third, we had no data available on the cause of death. It has been suggested that the mechanistic association between vitamin B12 and MMA can be disrupted in patients with advanced stages of malignant disease [19, 20]. Although we excluded subjects with known malignant disease at baseline, residual confounding by previously unrecognized malignant disease contributing to premature death during follow-up remains a possibility. A major strength of this study is that it is the first to investigate the prospective association of MMA levels with mortality. Additional strengths are the prospective study design, the large sample size, availability of MMA measurements, and follow-up data on mortality.

## Conclusions

In conclusion, we found that vitamin B12 and eGFR were significantly associated with MMA levels, but only explained a small part (i.e., 22%) of the variation in MMA values. In addition, we found that higher MMA levels are independently associated with an increased risk for mortality in a general population cohort. Moreover, we found a significant interaction between MMA, eGFR, and mortality, indicating that this association was more pronounced in individuals with impaired renal function.

## Supplementary Information

**Additional file 1 **Univariable and multivariable linear regression analyses for log_2_ MMA after exclusion of individuals that used multivitamin or vitamin B supplements (*n* = 1360).

**Additional file 2.** 3D plot depicting the unadjusted cross-sectional association between methylmalonic acid, vitamin B12 and eGFR after exclusion of individuals that used multivitamin or vitamin B supplements.

**Additional file 3.** Parallel comparison of prospective analyses of associations of log_2_ plasma total homocysteine, log_2_ MMA and their interactions with eGFR with respect to all-cause mortality.

**Additional file 4.** Prospective associations of log_2_ MMA, eGFR and of interaction of log_2_ MMA with eGFR, respectively, with all-cause mortality stratified for SES (n_events_ / n_total_ = 72/1533).

**Additional file 5.** Interaction of log_2_ MMA with eGFR and all-cause mortality after exclusion of individuals that used multivitamin or vitamin B supplements (n_events_ / n_total_ = 68/1360).

**Additional file 6.** 3D plot depicting the unadjusted interaction between methylmalonic acid and eGFR with all-cause mortality after exclusion of individuals that used multivitamin or vitamin B supplements.

## Data Availability

All data generated or analyzed during this study are included in this published article [and its supplementary information files].
