# Supplementary material for: Methylmalonic acid, vitamin B12, renal function, and risk of all-cause mortality in the general population: results from the prospective Lifelines-MINUTHE study
Source: BMC Med. 2020 Dec 10;18:380. doi: 10.1186/s12916-020-01853-x (PMC7726887; doi:10.1186/s12916-020-01853-x)
Supplement: Supplementary file 1 — Additional file 1 Univariable and multivariable linear regression analyses for log2 MMA after exclusion of individuals that used multivitamin or vitamin B supplements (n = 1360). [file 12916_2020_1853_MOESM1_ESM.pdf]

Additional file 1. Univariable and multivariable linear regression analyses for $\log_2$ MMA after exclusion of individuals that used multivitamin or vitamin B supplements (n = 1,360).

	Univariable		Multivariable Model 1		Multivariable Model 2	
	**Std b**	***P*-value**	**Std b**	***P*-value**	**Std b**	***P*-value**
Log_2 vitamin B_{12} (pmol/L)	-0.412	<0.001	-0.424	<0.001	1.510	0.02
Log_2 eGFR (mL/min/1.73 m^2)	-0.186	<0.001	-0.168	<0.001	0.906	0.01
Log_2 vitamin B_{12} x Log_2 eGFR	-	-	-	-	-2.215	0.004
**Demographics**						
Male sex	0.016	0.5	-0.039	0.1	-	-
Age (years)	0.091	0.001	0.051	0.046	0.038	0.1
High education	-0.078	0.004	-	-	-	-

Abbreviations: eGFR, estimated glomerular filtration rate; MMA, methylmalonic acid.
Model 1: R^2 = 0.21; Adjusted R^2 = 0.21.
Model 2: R^2 = 0.21; Adjusted R^2 = 0.21.
