# Supplementary figures and images for: Methylmalonic acid, vitamin B12, renal function, and risk of all-cause mortality in the general population: results from the prospective Lifelines-MINUTHE study
Source: BMC Med. 2020 Dec 10;18:380. doi: 10.1186/s12916-020-01853-x (PMC7726887; doi:10.1186/s12916-020-01853-x)

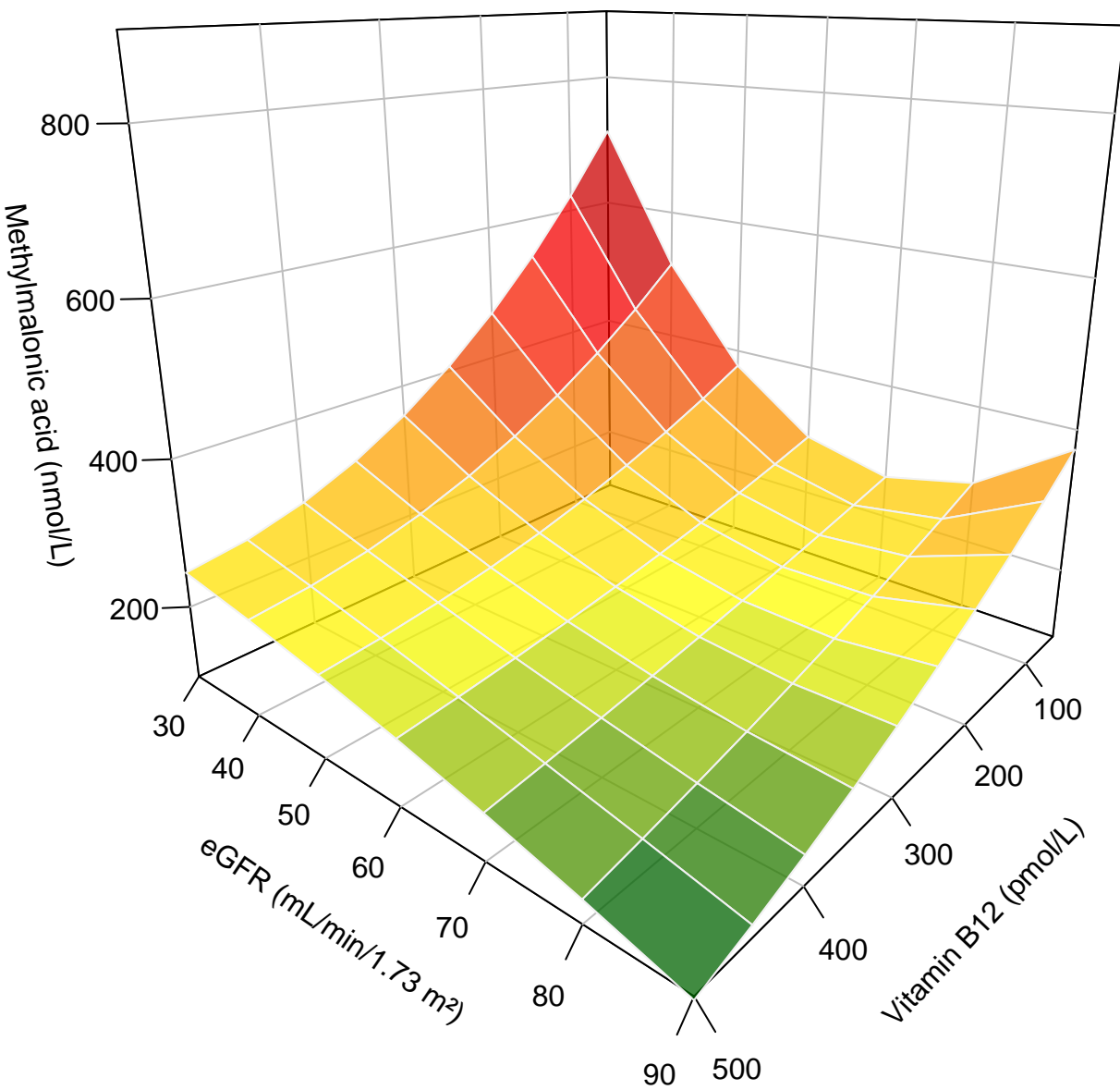

Supplement: Supplementary file 2 — Additional file 2. 3D plot depicting the unadjusted cross-sectional association between methylmalonic acid, vitamin B12 and eGFR after exclusion of individuals that used multivitamin or vitamin B supplements. [file 12916_2020_1853_MOESM2_ESM.pdf]

# Risk of mortality

Hazard Ratio

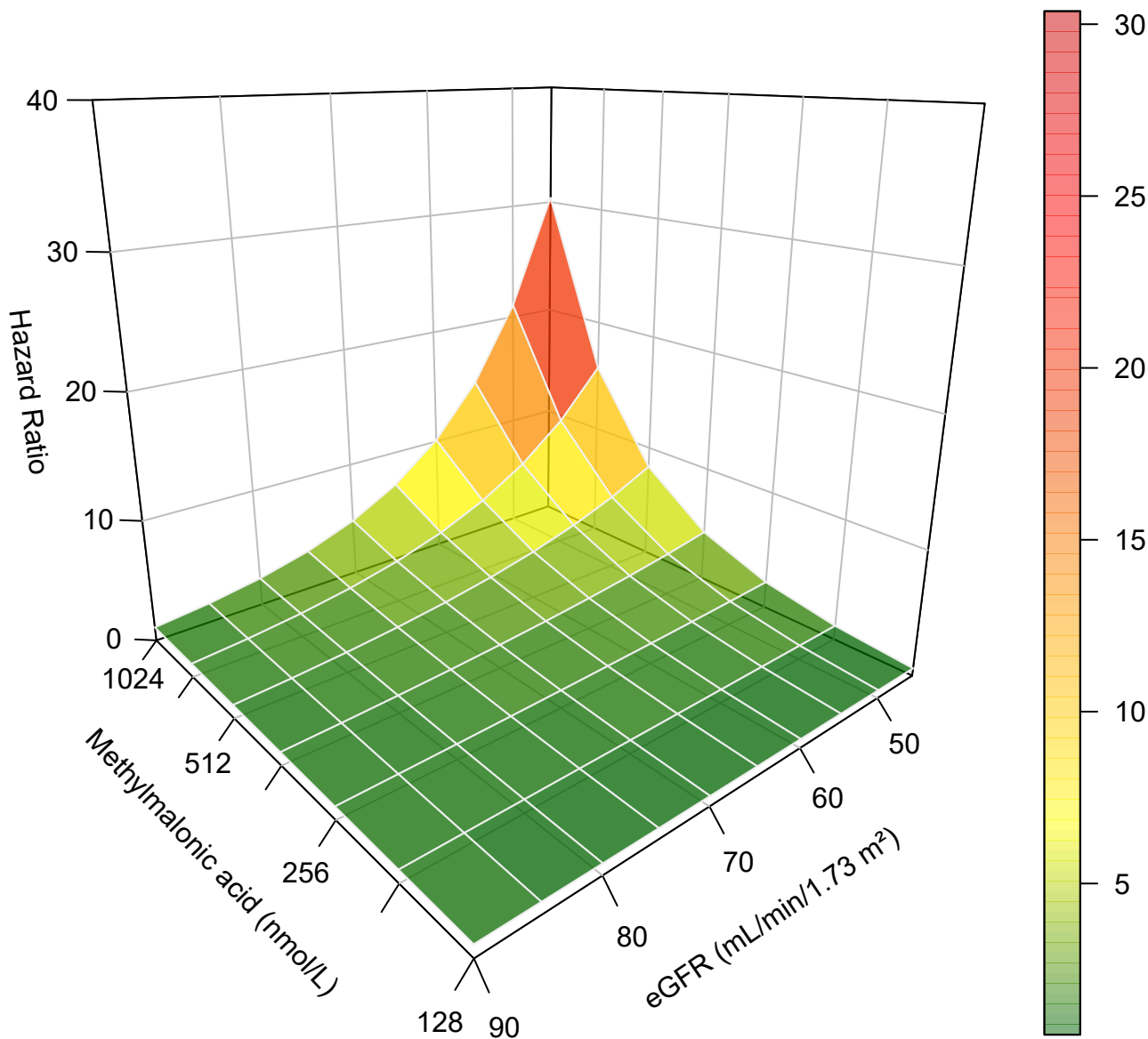

Supplement: Supplementary file 6 — Additional file 6. 3D plot depicting the unadjusted interaction between methylmalonic acid and eGFR with all-cause mortality after exclusion of individuals that used multivitamin or vitamin B supplements. [file 12916_2020_1853_MOESM6_ESM.pdf]
