# Supplementary material for: Methylmalonic acid, vitamin B12, renal function, and risk of all-cause mortality in the general population: results from the prospective Lifelines-MINUTHE study
Source: BMC Med. 2020 Dec 10;18:380. doi: 10.1186/s12916-020-01853-x (PMC7726887; doi:10.1186/s12916-020-01853-x)
Supplement: Supplementary file 3 — Additional file 3. Parallel comparison of prospective analyses of associations of log2 plasma total homocysteine, log2 MMA and their interactions with eGFR with respect to all-cause mortality. [file 12916_2020_1853_MOESM3_ESM.pdf]

Additional file 3. Parallel comparison of prospective analyses of associations of $\log_2$ plasma total homocysteine, $\log_2$ MMA and their interactions with eGFR with respect to all-cause mortality.

	**Models 1A and 1B**		**Models 2A and 2B**		**Model 3**	
	**HR (95% CI)**	***P*-value**	**HR (95% CI)**	***P*-value**	**HR (95% CI)**	***P*-value**
Homocysteine						
$\log_2$ homocysteine (nmol/L)	11.10 (3.38-36.45)	<0.001	7.80 (2.05-29.67)	0.003	2.24 (0.42-11.91)	0.4
eGFR (10 mL/min/m^2)	1.95 (0.92-4.12)	0.08	2.10 (0.91-4.84)	0.08	6.51 (1.72-24.69)	0.006
$\log_2$ homocysteine x eGFR	0.82 (0.69-0.97)	0.02	0.82 (0.68-0.99)	0.04	0.98 (0.77-1.26)	0.9
MMA						
$\log_2$ MMA (nmol/L)	13.33 (3.87-45.91)	<0.001	8.29 (2.34-29.34)	0.001	7.81 (1.59-38.23)	0.01
eGFR (10 mL/min/m^2)	7.36 (1.98-27.41)	0.003	6.51 (1.72-24.69)	0.006	6.51 (1.72-24.69)	0.006
$\log_2$ MMA x eGFR	0.76 (0.65-0.89)	0.001	0.79 (0.67-0.92)	0.003	0.79 (0.65-0.97)	0.03

Model 1: Two separate models with the terms $\log_2$ homocysteine, eGFR and $\log_2$ homocysteine x eGFR for homocysteine (model 1A) and the terms $\log_2$ MMA, eGFR, and $\log_2$ MMA x eGFR for MMA (model 1B, this is the same model as model 1 in table 3, and is included for comparison).

Model 2: Two separate models, both adjusted for age, sex, SES, smoking, alcohol intake, BMI, SBP, vitamin B12, use of vitamin supplements, with additional adjustment for $\log_2$ MMA in case of the model for homocysteine (model 2A) and with additional adjustment for $\log_2$ homocysteine in case of the model for MMA (model 2B).

Model 3: One overall model in which the terms $\log_2$ homocysteine, eGFR, $\log_2$ homocysteine x eGFR, $\log_2$ MMA, $\log_2$ MMA x eGFR are adjusted for each other and for age, sex, SES, smoking, alcohol intake, BMI, SBP, vitamin B12, and for use of vitamin supplements. Because it is one model, the eGFR term listed together with the terms for homocysteine is the same as the eGFR terms listed with the terms for MMA.

N_{events} / n_{total} = 72/1,533. Abbreviations: BMI, body mass index; eGFR, estimated glomerular filtration rate; SBP, systolic blood pressure; SES, socioeconomic status.
