# Supplementary material for: Methylmalonic acid, vitamin B12, renal function, and risk of all-cause mortality in the general population: results from the prospective Lifelines-MINUTHE study
Source: BMC Med. 2020 Dec 10;18:380. doi: 10.1186/s12916-020-01853-x (PMC7726887; doi:10.1186/s12916-020-01853-x)
Supplement: Supplementary file 4 — Additional file 4. Prospective associations of log2 MMA, eGFR and of interaction of log2 MMA with eGFR, respectively, with all-cause mortality stratified for SES (nevents / ntotal = 72/1533). [file 12916_2020_1853_MOESM4_ESM.pdf]

Additional file 4. Prospective associations of log_2 MMA, eGFR and of interaction of log_2 MMA with eGFR, respectively, with all-cause mortality stratified for SES (n_{events} / n_{total} = 72/1,533).

	**Model 1**		**Model 2**		**Model 3**	
	**HR (95% CI)**	***P*-value**	**HR (95% CI)**	***P*-value**	**HR (95% CI)**	***P*-value**
Log_2 MMA (nmol/L)	13.72 (3.96-47.50)	<0.001	16.35 (4.74-56.36)	<0.001	10.86 (3.14-37.62)	<0.001
eGFR (10 mL/min/m^2)	8.01 (2.12-30.16)	0.002	10.85 (2.85-41.28)	<0.001	7.20 (1.88-27.53)	0.004
Log_2 MMA x eGFR	0.75 (0.64-0.88)	<0.001	0.73 (0.63-0.86)	<0.001	0.77 (0.66-0.90)	0.001

Model 1: log_2 MMA, eGFR, log_2 MMA x eGFR.

Model 2: adjusted for age and sex.

Model 3: as model 2 + smoking, alcohol intake, BMI, SBP, vitamin B12 and use of vitamin supplements.

Abbreviations: BMI, body mass index; eGFR, estimated glomerular filtration rate; SBP, systolic blood pressure; SES, socioeconomic status.
