# Supplementary material for: Methylmalonic acid, vitamin B12, renal function, and risk of all-cause mortality in the general population: results from the prospective Lifelines-MINUTHE study
Source: BMC Med. 2020 Dec 10;18:380. doi: 10.1186/s12916-020-01853-x (PMC7726887; doi:10.1186/s12916-020-01853-x)
Supplement: Supplementary file 5 — Additional file 5. Interaction of log2 MMA with eGFR and all-cause mortality after exclusion of individuals that used multivitamin or vitamin B supplements (nevents / ntotal = 68/1360). [file 12916_2020_1853_MOESM5_ESM.pdf]

Additional file 5. Interaction of $\log_2$ MMA with eGFR and all-cause mortality after exclusion of individuals that used multivitamin or vitamin B supplements (n_{events} / n_{total} = 68/1,360).

	**Model 1**		**Model 2**		**Model 3**	
	**HR (95% CI)**	***P*-value**	**HR (95% CI)**	***P*-value**	**HR (95% CI)**	***P*-value**
Log_2 MMA (nmol/L)	12.63 (3.54-45.03)	<0.001	14.50 (4.10-51.20)	<0.001	10.47 (2.93-37.45)	<0.001
eGFR (10 mL/min/m^2)	7.40 (1.92-28.34)	0.004	10.05 (2.60-38.90)	0.001	7.27 (1.84-28.72)	0.005
Log_2 MMA x eGFR	0.76 (0.65-0.89)	0.001	0.74 (0.63-0.87)	<0.001	0.77 (0.65-0.91)	0.002

Model 1: $\log_2$ MMA, eGFR, $\log_2$ MMA x eGFR.

Model 2: adjusted for age and sex.

Model 3: as model 2 + SES, smoking, alcohol intake, BMI, SBP, vitamin B12 and use of vitamin supplements.

Abbreviations: BMI, body mass index; eGFR, estimated glomerular filtration rate; SBP, systolic blood pressure; SES, socioeconomic status.
